# Linking Supply Chain Governance and Biosecurity in the Context of HPAI Control in Western Java: A Value Chain Perspective

**DOI:** 10.3389/fvets.2018.00094

**Published:** 2018-05-02

**Authors:** Dikky Indrawan, Karl M. Rich, Peter van Horne, Arief Daryanto, Henk Hogeveen

**Affiliations:** ^1^Business Economics Group, Wageningen University & Research, Wageningen, Netherlands; ^2^East and Southeast Asia Regional Office, International Livestock Research Institute, Hanoi, Vietnam; ^3^Wageningen Economic Research, Wageningen University & Research, Wageningen, Netherlands; ^4^Business School, Bogor Agricultural University, Bogor, Indonesia; ^5^Department of Farm Animal Health–Epidemiology, Utrecht University, Utrecht, Netherlands

**Keywords:** value chain analysis, HPAI, chain governance, diversity of transactions, transaction cost economics, biosecurity

## Abstract

Despite extensive efforts to control the highly pathogenic avian influenza (HPAI), it remains endemic in Western Java, Indonesia. To understand the limited effectiveness of HPAI control measures, it is important to map the complex structure of the poultry sector. The governance of the poultry value chain in particular, could play a pivotal role, yet there is limited information on the different chain governance structures and their impacts on HPAI control. This article uses value chain analysis (VCA), focusing on an in-depth assessment of governance structures as well as transaction cost economics and quantitative estimates of the market power of different chain actors, to establish a theoretical framework to examine biosecurity and HPAI control in the Western Java poultry chain. During the research, semi-structured interviews were conducted with key value-chain stakeholders, and the economic performance of identified actors was estimated. Results indicated the co-existence of four different poultry value chains in West Java: the integrator chain, the semi-automated slaughterhouse chain, the controlled slaughter-point chain, and the private slaughter-point chain. The integrator chain was characterized by the highest levels of coordination and a tight, hierarchical governance. In contrast, the other three types of value chains were less coordinated. The market power of the different actors within the four value chains also differed. In more integrated chains, slaughterhouses held considerable market power, while in more informal value chains, market power was in the hands of traders. The economic effects of HPAI and biosecurity measures also varied for the identified actors in the different value chains. Implementation of biosecurity and HPAI control measures was strongly related to the governance structure of the chain, with interactions between different chains and governance structures accentuating the risk of HPAI. Our findings highlight that a proper understanding of the chain governance structure is vital to improve the effectiveness of HPAI control measures, by making the interventions more specific and fit-for-purpose given the incentive structures present in different chains.

## Introduction

Highly pathogenic avian influenza (HPAI) H5N1 is an important endemic disease in Indonesia (1, 2). HPAI outbreaks negatively affect public health but also food safety, social wellbeing and the broader economy. HPAI has been difficult to control in Indonesia for a variety of reasons. These include the limited capacity for pre-requisite programs that address HPAI prevention, limited disease surveillance activities, and low levels of public health regulation. Likewise, the Indonesian government has had difficulties implementing its planned HPAI control programs. For instance, vaccination at the farm level has not been effectively implemented (3), while surveillance activities for HPAI through the Participatory Disease Surveillance and Response program have had only limited success (4, 5). There were many cases of under-reporting of HPAI due to farmer fears for mandatory culling without proper compensation (6). Efforts to apply biosecurity measures in both the small-scale commercial (termed “sector 3” by the Food and Agriculture Organization (FAO) of the United Nations) and backyard (sector 4) poultry farms were largely unsuccessful. The proximity and mutual interaction of both types of smallholder poultry systems often reinforce disease dynamics and perpetuate recurrent “infection cycles” of HPAI (6).

An important underlying reason for the failure of HPAI control programs in Indonesia lies in the organizational and institutional structure of the poultry sector (7). The structure of rearing and selling poultry comprises all activities and interactions from farmer to consumer, and in Indonesia, that structure is complex with multiple links and interactions. Although the Indonesian poultry sector consists of a number of different value chains, there is a noteworthy lack of understanding of the structure of the existing value chains, the nature of value chain links and interactions, and how the poultry sector structure affects efforts to control HPAI. As noted by Rich and Perry (8), “weak” links in the chain can compromise control efforts at other stages, and as such, it is crucial to identify the incentives and pressures that drive these actors to work in “sub-optimal” ways from a disease control standpoint (even if economically rational). Therefore, understanding poultry value chain structures and their influence on HPAI control is important to develop incentives that drive chain actors to implement control measures. This can be achieved by employing value chain analysis (VCA) to analyze the marketing and governance structure of value chains.

An often overlooked aspect of the value chain is its governance structure, defined as the mechanisms that drive the coordination of transactions between actors. Value chains can be tightly governed through contracts or vertical integration where demands for quality or other product attributes are necessary. By contrast, transactions in traditional chains are simply governed by price and availability. Insight into the governance structure further reveals the power relations, which can be expressed in terms of diversity of transactions. When transactions are coordinated by a dominant chain actor, the ability of, or incentives for certain actors to comply with disease control will be affected.

Given our interest in linking VCA results to the control of HPAI, we used transaction cost economics (TCE) to relate governance to biosecurity practices (9). Which type of governance minimizes transaction costs depends on the relationship-specific investments (asset specificity) (9). Investments in biosecurity are one form of asset-specificity. In the case of HPAI control, these investments can be seen as risk mitigation practices that bind partners into tighter forms of coordination and improve incentives to control disease. In Indonesia, biosecurity investments and practices vary across different forms of value chain governance. Differences in biosecurity practices cause different risks of HPAI incursion within and between poultry chains. Moreover, where multiple types of value chain governance co-exist, there could be a variety of market and governance failures that spill over across different chains, driving the endemicity of HPAI. Since dominant actors may have a more significant role in the control of HPAI, we need to identify those actors that govern the chain. One approach to identify the dominant actor is by evaluating the chains’ economic performance and the distribution of profits over the various actors within the value chain (10). A proper understanding of the poultry value chain and its governance is vital to drive improved adoption of HPAI control strategies of different value chain stakeholders (8).

Research applying VCA in the context of animal diseases has emphasized the importance of the value chain perspective to evaluate livestock disease management strategies. VCA provides information on the flow of materials, resources, commodities, and value-adding activities between the different parts of the value chain (e.g., (7, 11–15). In the context of HPAI in Indonesia, research adopting a value chain perspective has been limited. Existing literature includes study chronicling the HPAI situation on Java (16); a case study of HPAI in Bogor (17); a qualitative risk assessment of HPAI (6); a study examining the alignment of poultry sector actors with avian influenza control in Indonesia (18); and a study identifying risk factors of HPAI (5). These research outputs from the International Livestock Research Institute (ILRI) and the FAO highlight the complexity of different poultry value chains in Indonesia, but do not provide an in-depth assessment of governance structures or the diversity of transactions with respect to HPAI control. Sudarman et al. (17) come closest in this regard, but their focus is more holistic, zooming on the chain rather than on governance as such.

The objective of this study is to assess the complexity of poultry value chain structures and their influence on HPAI control in Western Java, paying particular attention to the relationship between value chain structures, actors, governance, and economic performance. The study focuses on relations across different types of actors and does not explore the horizontal links within different chain nodes or public governance. The study provides an in-depth discussion of the poultry chain that explains critical control points for HPAI and where policy can more effectively intervene taking the complexity of the marketing chain into account. More detailed information about governance and transaction diversity in Western Java will improve our understanding of the poultry value chain, and the role governance plays in shaping economic motivations and behavior of value chain actors. Thus, such information can be used to incentivize all actors to participate in fit-for-purpose HPAI control strategies in Western Java.

## Analytical and Theoretical Framework

To understand the diversity of transactions and governance structures of the poultry value chain, we used three complementary approaches. We first performed a value chain analysis (VCA), following Kaplinsky and Morris( 2001), and applied it in an animal health context as in Rich and Wanyoike(11). This was followed by an analysis where we linked governance typologies to biosecurity practices (9, 10, 19). Finally, from the first two approaches, we derived quantitative estimates of economic performance (10). The results from these three approaches were combined to assess the risk factors of HPAI introduction and transmission, and the consequences of HPAI in the absence of government intervention.

First, VCA was used to construct the network of input-output relationships of the poultry supply chain. VCA tools allow practitioners to create a value chain map for the traditional and modern channels describing the actors and the nature of value chain governance structures. Value-chains represent the various processes involved in producing goods in the supply chain based on the notion of value-added at the production level. Once a value chain map has been identified, other approaches can be used together with VCA to obtain more insight into the poultry chain.

Second, governance structures were classified through the typology of Gereffi et al. (19). This typology illustrates the diversity of transactions triggered by the dominant actor’s needs, shifting the degree of coordination, the capabilities in the supply base, the ability to codify transactions and the complexity of transactions in the value chain. In this typology, Gereffi et al. (19) identified five types of governance structures based on the degree of transaction coordination between value chain actors. The most loosely coordinated mode of governance is through markets, i.e., on the basis of price and availability. A modular form of governance involves customization of a product by a seller to a buyer without any other form of explicit coordination. Relational governance involves transactions facilitated through specific relationships and mutual dependence between buyers and sellers (e.g., family ties). Captive governance typically involves the direct coordination of transactions by the buyer through contracts and the provision of inputs and technical support. Captive governance is often required when product specifications are exacting, necessitating tighter control of transactions by the buyer to ensure quality control. Finally, vertical integration involves transactions taking place solely within one organization or firm to ensure compliance with internal processes, rather than taking the risk of working with independent suppliers.

Using insights from TCE, we identified how different types of value chain governance patterns influence biosecurity practices. TCE helps to justify the rationale associated with different types of coordination (governance) mechanisms (20). The underlying assumption of the TCE approach is that the actors will choose the governance form that minimizes transaction costs. Three aspects of transaction cost underpin these decisions: the level of asset specificity, the level of uncertainty, and the frequency of transaction. Asset specificity refers to the degree of relationship-specific investments made by two parties to facilitate their transactions. Investments that are highly specific are unlikely to be productively re-used for other purposes, serving to bind actors more closely together. In such cases, tighter forms of coordination, such as contracts or vertical integration are required to protect those investments. Similarly, as the level of uncertainty (risk) and the transaction frequency (e.g., the intensity of exchange, number of times the same transactions take place) increase, greater coordination and tighter governance structures may be necessary. We posit that different types of biosecurity practices in different chains may be influenced by the coordination mechanisms associated with the governance structure of the value chain.

Third, we estimated economic performance via VCA to quantify the value added for each channel. Kaplinsky and Morris (10) define power as the ability of one party “to force other parties to take particular actions” or “to be deaf to demands of others”. Our power estimation used the value chain structure to estimate chain conduct in terms of price and quantity decisions. The estimated profits and the profitability were used as a measure of economic performance. Economic performance is an essential parameter to understand the pattern of returns as part of distributional outcomes in the value chain, showing the added value (output value minus input costs) for each link of the chain (10). The share of chain value added can be an indicator of a firm’s power, but qualitative indicators can be more relevant. Chain actors with a relatively high economic performance (profitability) can be seen as actors with a relatively high market power. They are able to exploit high prices and/or create barriers to entry (21). Knowledge about the share of chain value added can support other indicators that analyze power asymmetries such as the market structures (the number of buyers versus the number of sellers), the degree of dependence between buyers and sellers, and the characterization of the governance structures.

Finally, we assessed the risk factors of HPAI introduction and transmission and the consequences of failure to control HPAI in the chain. We looked at the enabling conditions generated under the different forms of value chain governance. Four factors can be used to identify the risk of HPAI introduction and transmission in relation with the value chain map, governance structure and the implementation of biosecurity: (1) the number of actors involved (22), (2) the frequency of contacts with a possible source (22, 23), (3) the number of links within the chain stages (13) and (4) the contact structure in the poultry chain (5, 13, 14, 22, 23). These four factors can be assessed based on the value chain map, the governance typologies present in each chain, and how they relate to the biosecurity practices in place.

## Materials and Methods

Table 1 shows the relation between the theoretical framework and the data collection process, and provides details on the specific actors interviewed during the study. We carried out three workshops, seven site visits and 26 in-depth interviews with several key value chain stakeholders, to assess the governance and biosecurity practices in the different identified poultry value chains. The data collected during the early phases of our research were validated in later steps. This enabled us to make a thorough assessment of governance in the poultry value chain, as compared to more conventional VCA studies. The interviews were based on semi-structured questionnaires. We specified the questions for the typology according to Gereffi et al. (19) on the degree of coordination, the capabilities in the supply base, the ability to codify transactions and the complexity of transactions. Questions regarding TCE were aimed at three aspects: the level of asset specificity, the level of uncertainty, and the frequency of transactions within each chain. We interviewed the respondents about biosecurity practices based on the FAO poultry biosecurity guidelines.

**TABLE 1:** Data collection and respondents.

**Approaches**	**Steps**	**Data Collected**	**Interviewed Actors**
Value Chain Map	Workshop 1 (focus group discussions) in December 2013	Actors Production systems Input, output, cost, price	4 high-level representatives of large integrated companies, the chairman of slaughter house association representative (ARPHUIN)
	Workshop 2 (focus group discussions) in December 2013	Actors Production systems Input, output, cost, price	2 representatives of a small semi-automated slaughterhouse in Bogor the chairman of the union of farmer association (GOPAN)
Value chain governance typology, TCE	Site visits in December 2013	Actors Production systems Biosecurity Chain governance TCE	1 poultry farm, 1 collecting farm, 1 integrator slaughterhouse, 2 semi-automated slaughterhouses, 1 slaughter-point/wet market, 1 specialty store
	In-depth interviews 1in January 2014	Actors Production systems Biosecurity practices Chain governance TCE	2 representatives of the banking sectors, 2 government officials, 2 representatives of farmer associations, 1 representative of traders 1 representative of a traditional private slaughter-point associations 1 integrator slaughterhouse, 2 semi-automated slaughterhouses
Value chain governance typology, TCE (validation)	In-depth interviews 2in January 2014	Actors Production systems Biosecurity practices Chain governance TCE	the Chairman of the Poultry Farmer Association (PINSAR) the Chairman of the Federation of the Indonesian Poultry Society (FMPI). 1 representative of academia
	In-depth interviews 3 inSeptember to November 2015	Actors Production systems Biosecurity practices Chain governance TCE	1 representative of the banking sectors, 2 government officials, 2 representatives of farmer associations, 1 representative of a traditional private slaughter-point associations 1 integrator slaughterhouse, 3 semi-automated slaughterhouses 2 specialty stores
Value chain economic performance (quantitative estimates of the market power)	Workshop 3 (focus group discussions) in March 2015	Actors Production systems Inputs per stage Outputs per stage Costs per stage Prices per stage Simulations	2 consultants 4 government officials 3 semi-automated slaughterhouses 1 representative automated slaughter house 1 representative of Farmer Associations (PINSAR) 1 representative of the union of farmer association (GOPAN)

The different workshops also provided information about (1) actor roles in coordination mechanisms such as the setting of product and process standards(for biosecurity and food safety); (2) the monitoring of performance, environmental standards, labor standards and conformance to ISO and HACCP standards; and (3) the different roles of actors in the implementation of sanctions whenever the performance of other actors within their chain does not meet the pre-specified requirements.

The key value chain stakeholders interviewed in this study were the Federation of the Indonesian Poultry Society (FMPI) (the only organization uniting all poultry actors in the region), the slaughter-house association (ARPHUIN) that represents the modern chain, the Union of Farmers Association (GOPAN) in Indonesia, the Poultry Farmers Association (PINSAR) representing the major group of farmers in Indonesia, the traditional private slaughter-point associations, and two government agencies (the agricultural agency and a regional office). We also interviewed other actors such as consultants and representatives from meat-specialty stores, the banking sector and academia.

The data were processed in five steps. First, the value chain map was drawn and completed with the number of actors. Subsequently, the map was classified based on the governance typology. Third, we calculated the economic performance of the governance structure using quantitative estimates. Fourth, we linked details on the value chain governance structure using TCE and the assessment of biosecurity practices. Lastly, we linked the governance structure with the economic consequences of HPAI in the absence of government intervention.

Quantitative estimates of the market power of different chain actors were based on enterprise budgets for each chain actor group by estimating costs of input, returns, and added value.

1. The output of Western Java poultry production was estimated based on the situation in 2013 using secondary data from the Agricultural Census (24). Total farm output was based on the number of broilers in three provinces: West Java, Banten, and DKI Jakarta.
2. The total farm output of Western Java was divided over the traditional and the modern channels. Since no exact information on the distribution of output over the chains was available, we made an estimation based on the focus group discussions and interviews. We assumed that farms in sectors 1 (industrial and integrated farms) and 2 (commercial poultry production with high biosecurity farms) served the modern channel and that farms in sectors 3 (commercial poultry production with low biosecurity farms) and 4 (village or backyard poultry farms) served the traditional channel. The output of these four farm types was distributed over the slaughterhouses and collecting points in their respective value chains.
3. For each actor group in the identified value chains, we calculated output in kilograms of poultry products based on the available knowledge on production size. Since weight was used as the unit of output, farm output was measured in terms of weight of delivered poultry, while slaughterhouse output depended on the carcass weight
4. For each actor, we calculated fixed costs, variable costs, and added value based on the situation in 2013, using secondary data from the Agricultural Census (24).
5. Revenues were calculated as the output of products multiplied by the product market price (average yearly price in 2013).
6. Finally, for each actor group, we calculated the profitability per chain stage (based on a cycle of production activity for farmers, and on a day of selling and production activities for collecting farms and slaughterhouses) by subtracting the costs from the returns. A cycle of production activity for a farmer refers to the growth cycle of poultry from day 1 until harvest.
7. All calculations were made in Indonesian Rupiah and then converted into Euro using the December 2013 exchange rate.
8. The results are presented as a comparison of total profit margin relative to the total turnover in a given chain. The total turnover was defined as the total sales revenue.

## Results

### Mapping the Poultry Value Chain

**Figure 1:**
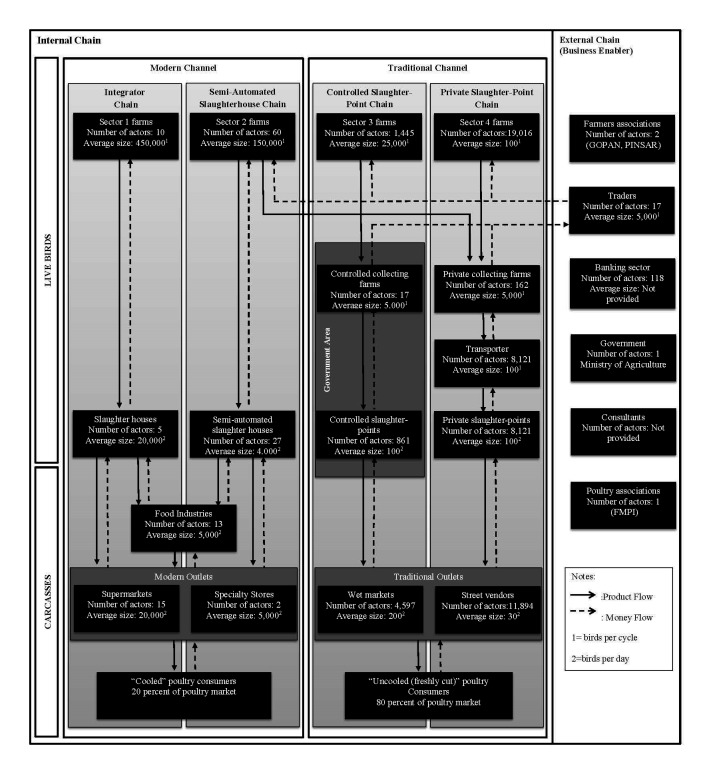
Mapping and approximate number and size (in birds per chain stage) of the actors poultry value chain in Western Java.

The analysis revealed two main marketing channels for poultry in West Java, which are illustrated in Figure 1. These channels were classified as the modern and traditional channels only serving the domestic market. The two marketing channels provide poultry meats with different characteristics. The modern channel produces cooled and frozen poultry meat, while the traditional channel produces freshly cut poultry meat without refrigeration or freezing. Therefore, these channels attracted different consumers with different preferences for poultry meat. Within these two channels, four specific chains could be distinguished: the integrator chain and the semi-automated slaughterhouse chain in the modern channel, and the controlled slaughter-point chain and the private slaughter-point chain in the traditional channel. Figure 1 illustrates the production and financial flows of the four different chains, and identifies the different links within and between the different value chains. The production flows are represented by the downward arrows, while the financial flows are represented by the dashed upward arrows. Stakeholders were characterized as internal or external actors, based on their involvement in the physical transport in the production flows. All stakeholders had both a direct and an indirect influence on the poultry transactions.

Most actors in the Western Java poultry chain were internal chain actors who are physically involved in the meat production and distribution, such as farms, collecting farms, transporters, slaughterhouses, slaughter-points, food processors and retails. These actors differed in number (Figure 1) and in their production characteristics. They were involved in transporting live birds and carcasses, using different transportation modes to end consumers. Live birds were produced at farms, and the mode of production depended on the farming system (sectors 1–4). The live birds from sector 1–2 that go to the modern channel were transported directly to the slaughterhouses, while the live birds from sector 2–4 that go to the traditional channel were transported through collecting farms. We noticed a relationship between sector 2 farms from the modern channel and the collecting farms from the traditional channel. Transport tools were owned by both slaughterhouses and collecting farms. Collecting farms are poultry shelters where live birds are brought together and sold. There are two types of collecting farms: controlled collecting farms and private collecting farms. The controlled collecting farms operate in a centralized government area, set up by the government to control the spread of HPAI. The government relocated many private collecting farms to a location owned by the government in order to control live bird movements. By contrast, private collecting farms operate in private locations or through home slaughtering. The average weight of live birds was 2.15 kg for sector 1, 1.5 kg for sector 2 and 1.3 kg for sectors 3 and 4. The average carcass weight by sector was 1.46 kg for sector 1, 1.13 kg for sector 2 and 0.98 kg for sectors 3 and 4.

Live birds were collected and processed in a slaughterhouse (automated or semi-automated) or slaughter-point (manual process), after which they were sold on the market. Live birds from private collecting farms that were to be slaughtered in private slaughter-points were transported by motorcycle. The transporters were informal actors, working part-time and receiving fees from the private slaughter points for their services. There were four types of slaughterhouse systems: the integrator slaughterhouse, the semi-automated slaughterhouse, the controlled slaughter-points, and the private slaughter-points. The integrator slaughterhouses consists of slaughter plants with modern equipment and holding HACCP, ISO, and state (NKV) certificates. The slaughter process at semi-automated slaughterhouses involves automated general stunning (water bath) and plucking, and transportation in shackles, but with all other work in the plant conducted manually. At controlled slaughter-points an actor that bought poultry from the controlled collecting farms, slaughters it in a centralized government area. Private slaughter-points are private houses in front of which workers slaughter poultry.

The total output was distributed in accordance to the focus group discussion results. The total output from sector 1 was distributed to the integrator chain. We assumed that the excess supply from sector 2 was distributed over the two chains in the traditional channel. Therefore, the higher quality output from sector 2 was distributed to the automated slaughterhouse (50%), while the lower quality was distributed to the controlled slaughter-point chain (5%), and the private slaughter-point chain (45%). Next, the output from sector 3 was distributed to the controlled slaughter-point chain (10%), and the private slaughter-point chain (90%). The total output from sector 4 was distributed to the private slaughter-point chain.

From the slaughterhouses, poultry meat was transported and sold to food processors, modern outlets such as supermarkets, and meat specialty stores. These outlets applied a cold chain and adhered to specific quality standards. Poultry meat from slaughter-points, however, was transported and sold through traditional channel outlets, such as wet markets and street vendors. These outlets sold fresh poultry meat using a temporary structure or mobile stall.

The transaction product flows in the internal chain differed across the modern and traditional chains. In the modern channel, the transactions were coordinated with rules and standards, while the traditional channel engaged in on-the-spot transactions, with low entry barriers but asymmetries in information among actors.

We identified a number of external actors that played a role in the value chain as business enablers, but were not necessarily physically involved in the production or distribution of poultry meat. One important example are traders at live bird markets. Traders are the individual actors between farmers and collectors. They play a critical intermediary role in terms of providing informal financial support in liaising transactions between farmers and collectors, and secondly they act as brokers matching farmers and collectors. Traders provide farmers with cash payments, and receive payments from collecting farms. This role started after the banking sector left the small and medium scale poultry business without support during the economic crisis of 1997. Transactions were based on the daily spot market, and there were no formal contracts or informal relations between traders and other actors. The banking sector provides business services such as the holding of financial assets and financial services for large companies, but far fewer services for farmers. There was no direct involvement from the banking sector to support investments to control HPAI. A number of organizations worked together with the government to address HPAI. PINSAR and GOPAN are the poultry farmer associations that advocate and support farmers, while ARPHUIN is the slaughterhouse union. FMPI is a poultry federation that facilitates communication and advocates for the poultry business on behalf of all poultry actors. The government plays a role in the food safety system to control the transmission of HPAI in the poultry sector production and market. Independent consultancy companies also played a role in the system through the provision of expert advice on the poultry business or on food safety in the modern channel, for example regarding ISO standards and HACCP certification.

### Governance Structures in the Poultry Value Chain

We found a wide range of governance structures in the different poultry value chains. Based on the typology of Gereffi et al. (19), we observed the presence of a hierarchy type governance in the integrator chain, modular governance in the semi-automated slaughterhouse chains, and market governance in the controlled slaughter-point and private slaughter-point chains. The other two typologies, the relational and captive governance structure, were not identified in these chains (Table 2).

**TABLE 2:** Types of value chain governance in the poultry meat value chain of West Java.

**Chain governance structure determinants (Diversity of transactions Criteria)**	**Modern channel**		**Traditional channel**	
	** Integrator chain**	** Semi-automated slaughterhouse chain**	**Controlled slaughter-point chain**	** Private slaughter-point chain**
	***Hierarchy***	***Modular***	***Market***	***Market***
Degree of coordination	High	Low	Low	Low
Capabilities in the supply base	Low	High	High	High
Ability to codify transactions	Low	High	High	High
Complexity of transactions	High	High	Low	Low

In the hierarchical form of governance in the integrator chain, slaughterhouses acted as the lead firms with explicit coordination of the other actors in the chain. This chain was vertically integrated, employing full managerial control to produce products in-house. The level of coordination between actors was high because of the complexity in the requirements for meat quality, including standards for cold and frozen products, size/weight, biosecurity, halal certification, NKV certification, HACCP certification, and ISO certification. Only NKV and HACCP certification standards induced the slaughterhouse as the leader of the chain to control HPAI. These certificates are required for doing business in this chain. Prices and volumes were arranged via material requirement planning to ensure timely supply.

The modular form of governance was found in the semi-automated slaughterhouse chain, where suppliers had a responsibility to make products or provide services to meet customer expectations. For instance, farmers needed to meet buyer requirements with regard to size, weight and on-farm biosecurity (e.g., isolation, traffic control and sanitation), and the semi-automated slaughterhouses had to provide a product specified by the retailers. In this chain, no private or public standards induced the slaughterhouse as the leader of the chain to control HPAI. A form of contract was used, but the buyer-supplier interactions were limited to the delivery specifications and prices and not via specific, long-term coordination. The traditional channels were characterized by market governance. In these two value chains, transactions were relatively simple, with no formal cooperation between actors. These channels had a low mutual dependence related to reputation, or family and ethnic ties between actors in vertical chain stages. The buyers provided suppliers with limited or no information about product specifications. We found diseased poultry was sold in these chains, therefore we labeled them as a “sick” poultry market. Traders had a relatively larger role coordinating the chain as external actors.

### The Economic Performance of the Poultry Value Chain in West Java

**Figure 2:**
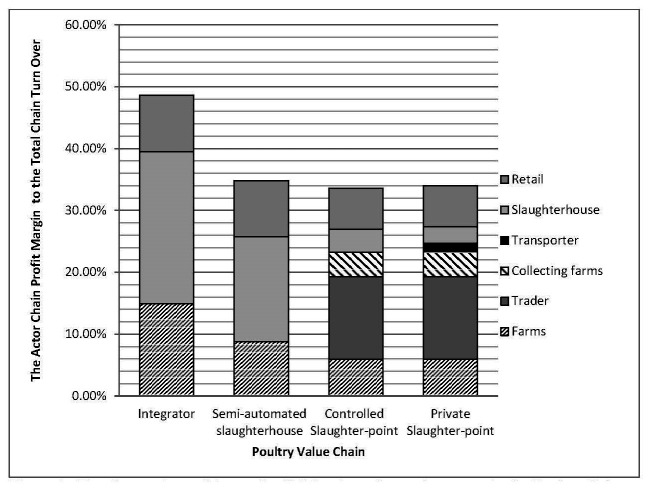
The Comparison of Actors Profit Margin to the total turnover in the Poultry Value Chain. The graphic bars represent the joint profit margin contributed bv each actor groups to total turnover (total sales revenues) in different chain governance. Each block in the graphic bars represents each actor group profit margin to the total turnover.

As illustrated in Figure 2, we computed economic performance at chain level as the share of the total profit margin relative to the total turnover in a given chain. We found that the integrator chain had the highest economic performance, because the share of the total profit margin relative to the total turnover was the highest (Figure 2). The other three chains had similar but lower profit margins.

If we look at the distribution of profit within and across the different groups, a number of interesting results emerge. In the modern channels, slaughterhouses had a higher share of the profit margin than farmers and retailers. By contrast, in the more traditional channels, the total profit margin was distributed over more actors, with the largest share captured by the traders. The comparison of actor profit margins within the different chains may illustrate the power of a specific chain actor. In this case, the slaughterhouse seemed to have the highest power in modern channels, while in traditional channels, the highest power was held by traders. Consequently, those who had market power were acting as the chain leader and had the largest influence on chain governance. Indeed, the presence of only a handful of traders compared to the significantly larger number of other actors (Figure 1), suggests a form of oligopolistic power held by traders in the traditional channel. The ability of slaughterhouses and traders to drive the value chain is the key determinant to impose biosecurity standards and control HPAI in all forms of chain governance.

### Biosecurity

We looked at the role of chain governance in the application of biosecurity practices for the four different value chains we identified. We assumed that differences in chain governance influence the risk of HPAI transmission (13). In this context, we took a transaction cost economics approach to test our hypothesis as to whether more coordinated chains lead to more investments in biosecurity practices. We differentiated two aspects of biosecurity: (1) the risk of disease introduction, and (2) the risk of disease transmission. The risk of HPAI introduction is the likelihood that the virus enters the value chain, for instance from another value chain. The risk of HPAI transmission is the likelihood of HPAI being transmitted within the value chain, for instance from one stage to the next after introduction of the virus.

Table 3 summarizes the biosecurity practices for each value chain type. While three transaction characteristics were observed, the level of asset specificity was most strongly related to the application of biosecurity practices. The other characteristics of transactions, uncertainty and frequency, were not considered by the actors as drivers for the application of biosecurity measures. Inciting suppliers to adopt biosecurity practices could be a way to mitigate uncertainty in the supply chain. Strong hierarchies and tight coordination amongst actors within the integrator chain facilitated a variety of specific investments, including those on biosecurity. These included maintaining biosecurity through a compartment system at the farm level (among section 1 farms), while slaughterhouses had stringent quality processes, which were HACCP, ISO and NKV certified. In a well-coordinated value chain, such as for the hierarchical form of governance, it was easier to implement and maintain biosecurity practices and, therefore, well-coordinated chains were better protected against HPAI introduction and transmission.

**TABLE 3:** Biosecurity practices and governance forms in the poultry value chains of West Java.

**Chain Governance Structure determinants (TCE Criteria)**	**Modern Channel**		**Traditional Channel**	
	** IntegratorChain**	** Semi-automatedSlaughterhouseChain**	**Controlled Slaughter-point Chain**	** Private Slaughter-point Chain**
	***Hierarchy***	***Modular***	***Market***	***Market***
Level of Asset Specificity	High	Medium	Low	Low
Level of Uncertainty	Low	High	High	High
Transaction frequency	Low	High	High	High
**Biosecurity Practices**	*High*	*Medium to low*	*Low*	*Low*

Asset specific investments in the semi-automated slaughterhouse chain (modular governance) were lower than in the integrator chain, because a form of contract (limited to price and weight specifications with general disease status) was used to support transactions within this chain. While contracts between actors included product specifications with regard to disease status, there were no efforts to support the supplier to increase biosecurity in order to fulfill these requirements. Therefore, biosecurity measures were limited in this chain and depended on the efforts of each individual actor to fulfill the contract requirements. Because of the low level of asset specificity in the semi-automated chain, chain actors were able to trade more freely with other partners, increasing the scope of the transaction but with less coordination. Consequently, investments to promote biosecurity were lower. In this chain, sector 2 farms even traded live birds with collecting farms in traditional channels where biosecurity practices were much lower still. As the risk of HPAI introduction in the controlled and private slaughter-point chains was higher than in the semi-automated slaughterhouse chain, sector 2 farms were at relatively high risk of introducing HPAI in their value chain (Figure 1). Improving coordination in the semi-automated slaughterhouse chain and cutting off trade with the controlled and private slaughter-point chains would most likely have a large effect on overall HPAI incidence in this chain.

There were no relation-specific investments in traditional channels. Transactions in these chains were based on price and convenience, in the absence of specific biosecurity requirements or coordination. Sick poultry was traded in these channels, and a sick poultry market was established that was also used by the semi-automated chain upon HPAI occurrence. The intensity of physical exchange and thus the risk of HPAI transmission was high. In the controlled slaughter-point chain, limited levels of biosecurity were applied in the sector 2 farms, that also delivered to the collecting farms. The majority of the live birds that came from sector 3 farms were mixed with those of sector 4 farms which applied only a minimal level of biosecurity. No biosecurity measures were applied in the collecting farms, during transport, or at the private slaughter-points.

Other researchers have shown that the type of governance affects actor perceptions of the importance of biosecurity (14, 23). This difference in perception influences the implementation of biosecurity practices and hence the risk of HPAI introduction and transmission in the different poultry value chains. We assessed this influence based on the four factors that affect the risk of HPAI introduction and transmission in relation to the value chain map, governance structure and the implementation of biosecurity. As shown in Table 4, each of the four factors that influenced the risk of HPAI introduction and transmission had a stronger effect in the less coordinated chains. This means that the risk of introduction and/or transmission of HPAI was much higher in the traditional channels as compared to the modern channels. Moreover, the links and contacts between the semi-automated chain and traditional channels created an additional layer of risk of disease transmission. Therefore, the integrator chain provided better protection against HPAI outbreaks as compared to chains with other forms of chain governance.

**TABLE 4:** Risk factors of HPAI introduction and transmission in different poultry value chains in West Java.

**Enabling condition of HPAI introduction and transmission in the chain governance**	**Modern Channel**		**Traditional Channel**	
	** Integrator chain**	** Semi-automated slaughterhouse chain**	**Controlled slaughter-point chain**	** Private slaughter-point chain**
	***Hierarchy***	***Modular***	***Market***	***Market***
1. Number of actors involved	+	+ +	+ + +	+ + + +
2. The frequency of contact	+	+ +	+ + +	+ + + +
3. Number of links in chain stages	+	++	+ +	+ + +
4. Contact structure	+	+ + +	+ + +	+ + + +
**Total risk of HPAI**	**+**	**+ +**	**+ + +**	**+ + + +**

Note, + = the least likelihood of risk, + + + + = the highest likelihood of risk

A TCE perspective highlights that many routes for disease transmission in the value chain were mediated at least in part by investments in biosecurity that arise from the types of governance that exist in the value chain. We found an important risk of backward transmission, e.g., from the markets to the farms or from the slaughterhouses to the farms. Crates and other materials used to transport poultry could act as vectors in the transmission of HPAI. Slaughterhouses were indeed reported to be associated with HPAI outbreaks (25). Poor biosecurity practices at the collecting farms, slaughterhouses, and slaughter-points could lead to infection of farms through interactions between humans, vehicles and crates, especially during the process of returning poultry crates from the market or the slaughterhouses to farms. In order to decrease the risk of introduction or infection in the less coordinated chains, the chain leaders (traders) would need to invest in more formal relationships that include biosecurity requirements, since traders were the only actors with the financial and management capabilities to invest in new production assets. This means that traders should upgrade their role from informal financers of the transaction into more formalized commercial agents, such as financial institutions or collecting farms. This could reduce the number of “infection cycles” in the complex and poorly-coordinated poultry chains. However, traders have no incentive to do so, as improved biosecurity practices do not affect their profits. Indeed, removing the “sick poultry market” would rather reduce trader profitability. This is in contrast with the chain leaders in the modern channels (slaughterhouses) who have incentives (economic performance to protect) to maintain improved biosecurity practices in their chains.

### The Economic Consequences of HPAI in Different Poultry Value Chains

The economic consequences of HPAI were influenced by the biosecurity practices in the value chains (23, 25, 26). HPAI incidents increased the mortality rate of poultry. Hence, the number of live birds that could be sold was reduced. In theory, a lower supply of poultry, will lead to increased prices at the farm gate in all value chains, and eventually the retail price will increase as well. We assessed these consequences based on the information gathered for the production related to disease incidents, including inputs, outputs, and prices per stage in different chains. Our research, however, indicated different consequences of HPAI incidents in the different value chains. The consequences of HPAI incidents in different identified poultry value chains in Western Java are illustrated in Table 5.

**TABLE 5:** Consequences of HPAI without government intervention.

**Consequences types (Losses)**	**Modern Channel**		**Traditional Channel**	
	** IntegratorChain**	** Semi-automatedSlaughterhouseChain**	**Controlled Slaughter-point Chain**	** Private Slaughter-point Chain**
	***(Hierarchy)***	***(Modular)***	***(Market)***	***(Market)***
Production	**+ + + +**	**+ + +**	**+ +**	**+**
Farm Price Effect	**+ + + +**	**+ + +**	**+ +**	**+**
Retail Price Effect	**+ + + +**	**+ + +**	**+**	**+**
**Overall**	**+ + + +**	**+ + +**	**+ +**	**+**

Note, + = the least likelihood of consequences, + + + + = the highest likelihood of consequences

In the most coordinated chain (integrator chain) HPAI incidents had the most severe consequences (Table 5). Because of the biosecurity practices in place, farms were forced to remove sub-clinically infected poultry from their flocks. The removal caused shortages in the supply of live birds (high quality poultry) to the slaughterhouses. Consequently, given the larger volumes traded by integrators, such shortages affected the price at the farm gate and ceased production at the slaughterhouse. The subsequent shortage in meat supply would increase prices at the retailer level. Thus, the reduction in activity would reduce profitability within this chain.

In less coordinated chains, the consequences of HPAI incidents were less severe (Table 5). For those actors ordinarily selling to formal markets, incidents of HPAI allowed actors to switch sales to the traditional channel (14), making these chains more resilient to fluctuations in the supply of poultry, but also more prone to new HPAI occurrences. Indeed, the private slaughterhouse chain assisted farmers in the semi-automated slaughter chain to trade their sub-clinically infected poultry. Therefore, during an HPAI outbreak, farmers under modular and market forms of governance (sector 2, 3 and 4 farms) were able to sell their poultry to the sick poultry market and thus mitigate the economic consequences of HPAI at a nodal level. However, the ability to trade across channels depended on the size of the outbreak. When HPAI outbreaks were large, farms in the semi-automated slaughterhouse chain were unable to supply enough poultry to the semi-automated slaughterhouse. In these cases the semi-automated slaughterhouses saw a decrease in production, affecting the farm price of poultry.

In general, the overall consequences of HPAI in situations where market governance prevails were lower than in situations of hierarchy and modular governance. The existence of a sick poultry market in this chain partially mitigated the production consequences of HPAI, leading to smaller effects on farm and retailer prices. Because consumers Any doubtin traditional channels were less informed about the quality of the product, retailers could sell sick poultry, having only to accept a slightly lower price.

## Discussion

In this study, we carried out an extensive value chain analysis, paying much attention to the governance structures in the Western Java poultry system. Our results indicate that the economic consequences of an HPAI outbreak vary for different governance structures. In particular, chains that were more tightly coordinated had more incentive to implement HPAI control measures compared to traditional channels. Therefore, the risk of HPAI introduction and transmission was lower.

Like all value chain studies, our approach was limited by its sampling frame. We implemented a convenience sampling framework for the different actors, given the complexity of value chains and the difficulties in obtaining representativeness among certain types of actors, particularly traders, wholesalers, and processors. Rich and Wanyoike (11) used a similar approach to extrapolate the broader value chain impact of Rift Valley Fever in Kenya. Although a larger sample would allow for a more detailed quantitative validation, our goal was to offer a qualitative view of the governance structure of the poultry sector. Moreover, resource constraints limit the ability of practitioners to carry out extensive informant-based data collection. Therefore, a relatively limited number of semi-structured interviews with key informants is justified for this study.

A number of issues with regard to the effectiveness of HPAI control measures and how they are related to governance can be identified. First, the effectiveness of HPAI control measures depends on the removal of the sick poultry market from the poultry chain. Without this, efforts to control HPAI will not be effective, since the existence of this market has largely removed the economic motivation of farmers and other actors to improve biosecurity. Traders will need to included, but it is unclear whether they will have the economic incentives to cooperate. Without this intervention, motivating actors, upstream and downstream in the chain will be difficult. Second, because of their higher risk of disease introduction and transmission as well as the limited economic incentives to prevent and control outbreaks, government interventions should focus on the less coordinated chains. Nonetheless, more moderately coordinated chains (e.g., the semi-automated chain) should receive particular attention, as they sell to both formal and informal markets, presenting a greater transmission risk. Third, the value chain map shows that traders play an important role as external actors in HPAI transmission. Analyzing chain governance shows that traders have an important decision-making role regarding the distribution of sick poultry to the market. Many control measures did not involve the participation of traders; therefore sick poultry markets have remained viable. Government intervention should aim at upgrading the role of traders from informal to formal commercial agents, such as financial institutions or collecting farms.

Existing coordination mechanisms have resulted in a lack of effective interventions within the traditional poultry sales channels, and improving coordination could lead to better HPAI control. Higher levels of coordination are correlated with improved application of biosecurity measures, thus strengthening coordination will likely reduce the risk of HPAI introduction and transmission in the poultry chains (13). Contracts can be implemented, as one form of a coordination mechanism, to improve meat quality and actor revenues (14). In turn, contract farming would create incentives to increase biosecurity and remove the sick poultry market.

On the other hand, increased coordination comes with higher transactions costs. It is not clear whether chain actors possess either the supply-side or demand-side incentives to address coordination without some form of government intervention or regulation. In order to create effective incentives, an intervention policy tailored to different value chains will be required (8, 14). For the situation in Western Java, a pull-and-push strategy could be applied. This approach uses economic performance as the main driver to stimulate chain actors to invest in improved coordination mechanisms and to produce healthier, higher quality poultry. The push strategy attempts to give greater incentives to chain actors in poultry production. Such incentives can take the form of bottom-up entrepreneurial support to encourage change (financial, organizational or technical incentives, or market access, property rights, and contracts) or a more direct, top-down regulatory approach. Neither approach is mutually exclusive, and indeed a combination of incentives and penalties is crucial where transactions costs and information asymmetries are high (27). The pull strategy is an incentive mechanism that is aimed at consumers. The pull strategy would try to convince consumers to change their preference towards higher quality poultry. Changes in consumer buying preferences would drive actors in poultry chains to meet consumer demand by improving the coordination of transactions (asset specific investments). Both intervention strategies are costly, therefore proving that the regulatory cost of improving standards and controls is a major consequence of HPAI.

## Conclusions

An extensive value chain analysis showed that four different poultry value chains can be distinguished in Western Java: the integrator chain, the semi-automated slaughterhouse chain, the controlled slaughter-point chain and the private slaughter-point chain. These four chains differ in structure, number and size of actors, economic performance, and in governance mechanisms. Moreover, the effects of HPAI vary in the different value chains as well as the effectiveness of HPAI control measures.

A close relationship was found between the poultry chain structure, coordination mechanisms, and the risk of HPAI introduction and transmission. First, the relationship between poultry chain structure and chain governance influences the effectiveness of current HPAI control measures. Second, the diversity in governance implies that there is no “one-size-fits-all” strategy for HPAI control measures that can be applied across different poultry value chains. Third, there are fewer economic incentives in less-coordinated chains (traditional channels) to participate in HPAI control programs. This means that in order to improve the HPAI situation in Western Java, it would be advantageous if government intervention improved incentives for better coordination of the different value chains. Improving the institutional infrastructure is a crucial condition for HPAI control to be effective.

## Author Contributions

DI designed the study, collected and analyzed the data, and drafted the manuscript. The remaining authors provided input on the design of the study, helped interpreting study results, and critically revised the manuscript. All authors read and approved the final manuscript.

## Conflict of Interest Statement

The authors declare that the research was conducted in the absence of any commercial or financial relationships that could be construed as a potential conflict of interest.
